# Knockdown of KCNQ1OT1 Inhibits Proliferation, Invasion, and Drug Resistance by Regulating miR-129-5p-Mediated LARP1 in Osteosarcoma

**DOI:** 10.1155/2020/7698767

**Published:** 2020-09-04

**Authors:** Yunfei Zhang, Wei Cai, Yuli Zou, Hong Zhang

**Affiliations:** ^1^Department of Pathology, Affiliated Hospital of Northwest Minzu University, Second Provincial People's Hospital of Gansu, Lanzhou, 73000 Gansu Province, China; ^2^Department of Pathology, People's Hospital of Gansu Province, Lanzhou, 73000 Gansu Province, China; ^3^Department of Emergency, Affiliated Hospital of Northwest Minzu University, Second Provincial People's Hospital of Gansu, Lanzhou, 73000 Gansu Province, China

## Abstract

KCNQ1OT1 exerts an important role in various cancers, but its role in osteosarcoma (OS) and the potential mechanism remain to be clarified. In the present research, we aimed to explore the effect of KCNQ1OT1 on osteosarcoma and further explore the special molecular mechanism. The expression of KCNQ1OT1 was analyzed in tumor and adjacent tissues of 30 patients with osteosarcoma by RT-PCR. Cell proliferation and invasion were explored using MTT and transwell assay, respectively. Luciferase reporter analysis and pull-down assay were performed to determine the binding activity of KCNQ1OT1 and miR-129-5p. The result revealed that KCNQ1OT1 was highly expressed in osteosarcoma tissues and cells. KCNQ1OT1-siRNA inhibited the proliferation, invasion, and drug resistance of osteosarcoma cells. The luciferase reporter assay and pull-down assay demonstrated that KCNQ1OT1 directly interact with miR-129-5p. In addition, miR-129-5p binds to LARP1 directly, and LARP1 promoted the proliferation, invasion, and drug resistance of osteosarcoma cells. What is more, KCNQ1OT1 promoted proliferation, invasion, and drug resistance via inhibiting the expression of miR-129-5p and further promoting the expression of miR-129-5p-mediated LARP1. Collectively, it suggests that downregulation of KCNQ1OT1 inhibits proliferation, invasion, and drug resistance by regulating miR-129-5p-mediated LARP1 in osteosarcoma cells.

## 1. Introduction

Osteosarcoma (OS) is a highly malignant neoplasm predisposing to children and adolescents, which is accounting for about 5% of children's tumors [1]. Clinically, most of the OS patients are males, and the ratio of males to females is about 1.5 : 1 [2]. According to statistics, the incidence of osteosarcoma is increasing year by year. The mortality rate of cancer patients under 20 years old is as high as 10% [3]. At present, the main treatment of osteosarcoma is chemotherapy. However, the drug resistance of osteosarcoma cells becomes more and more serious with the extensive use of drugs [4]. Therefore, it is necessary to study the drug resistance of osteosarcoma cells.

In many tumors, lncRNAs regulate gene expression related to abnormal proliferation and chemotherapy resistance [5]. KCNQ1OT1 (also known as LIT1), KCNQ1 opposite strand/antisense transcript 1, regulates the expression of neighboring genes through epigenetics [6]. Previous studies have shown that KCNQ1OT1 is upregulated in colon cancer and is therefore considered to be a tumor marker [7]. Similarly, the overexpression of KCNQ1OT1 promotes the occurrence and development of liver cancer [8]. However, the expression and role of KCNQ1OT1 in osteosarcoma need to be further elucidated.

MicroRNAs (miRNAs) are evolutionarily conserved noncoding RNA and decreased the target gene expression after transcription [9]. Recently, more and more reports indicate that miRNA plays a key role in the development and survival prediction of cancer by regulating cell proliferation, invasion, and drug resistance [10]. It has been demonstrated that miR-129-5p is an important tumor suppressor [11]. The overexpression of miR-129-5p significantly reduces the viability of cancer cells, including gastric, lung, and uterine cancers, lung adenocarcinoma, malignant glioma, and chondrosarcoma [11, 12].

La-related protein 1 (LARP1) is a highly evolutionarily conserved RNA-binding protein (RBP), belonging to the LARP family [13]. LARP1 has recently been shown to play an important role in the mTOR signaling pathway by combining with RAPTOR [14]. The LARP1 protein is highly expressed in various malignant tumors and is a predictor of adverse prognosis in cancer [15, 16].

In this study, we explored the effect of KCNQ1OT1 on the development of osteosarcoma and its potential mechanisms. Results suggested that the knockdown of KCNQ1OT1 inhibited the cell proliferation invasion and drug resistance in osteosarcoma, and miR-129-5p was a target of KCNQ1OT1. In addition, LARP1, as the target gene of miR-129-5p, promoted the proliferation, invasion, and drug resistance of osteosarcoma cells. Therefore, the downregulation of KCNQ1OT1 inhibited proliferation, invasion, and drug resistance by regulating miR-129-5p-mediated LARP1 in osteosarcoma.

## 2. Materials and Methods

### 2.1. Tissue

From October 2017 to December 2018, 30 patients with osteosarcoma were treated, including 18 males and 12 females, aged 5-16 years (average 8 years). Pathological examination confirmed the diagnosis. The patients were treated in our hospital. In addition, fresh tumor tissue samples were collected from all these patients, and matched adjacent tissue samples were taken as controls. The research was approved by the local Ethics Committee, and written consent of all selected patient guardians was obtained.

### 2.2. Cell Lines

The cell lines HFOB1.19, HOS, MG63, 143B, and U20S were purchased from Cell Applications (San Diego, CA, USA). The cells were cultured in DMEM medium containing 10% FBS and stored in a humidified incubator of 5% CO_2_ at 37°C.

### 2.3. Reagents

The reagents used were the following: DMEM medium and FBS (Gibco, Carlsbad, CA, USA); RNA extraction kit, reverse transcription kit, and RT-PCR kit (Beijing Full-style Gold Biotechnology Co., Ltd., Beijing, China); primer synthesis (Beijing Engine Biotechnology Co., Ltd., Beijing, China); and CCK-8 kit, protein quantitative kit, and fine cell lysate (Shaanxi Sanli Biotechnology Company, Xi'an, China). Antibodies including anti-LARP1, anti-P-gp, and anti-MRP1 were all obtained from Invitrogen Biotechnology Co., Ltd. (Massachusetts, USA). GAPDH was purchased from Abcam Inc. (Cambridge, UK).

### 2.4. Transfection

KCNQ1OT1-siRNA, control-siRNA, pcDNA3.1-LARP1, and vector pcDNA3.1 were designed and synthesized by Tsingke Biotech Co., Ltd. (Beijing, China). The plasmid vectors were transfected into U20S cells using Lipofectamine 2000 (Invitrogen, USA) according to the manufacturer's instructions.

### 2.5. MTT Assay

To determine the IC50 of liver cells for 5-Fu, the MTT assay was performed. Cells were transfected with KCNQ1OT1-siRNA or pcDNA3.1-LARP1, respectively, and 5-Fu (100 *μ*g/mL) was added at 24 h posttransfection. Then, 40 *μ*L of MTT diluted in 200 *μ*L of culture medium was added to each well of the 96-well culture plates. The cells were incubated for 1 h, and the absorbance of each well at 570 nm was recorded. We followed the methods of Yan et al. [17].

### 2.6. Luciferase Reporter Assay

Two luciferase reporters containing WT LARP1 (WT-LARP1) or MUT-LARP1 were generated to analyze the interaction between LARP1 and miR-129-5p. MUT-LARP1 contained several mutation sites (MUT-LARP1) that abolishes targeting by miR-129-5p. In addition, two luciferase reporters containing WT-KCNQ1OT1 (WT-KCNQ1OT1) or MUT-KCNQ1OT1 were generated to analyze the interaction between KCNQ1OT1 and miR-129-5p. MUT-KCNQ1OT1 contained several mutation sites (MUT-KCNQ1OT1) that abolish targeting by miR-129-5p. The miR-129-5p mimic or NC luciferase activity was determined by the luciferase analysis system (Promega, USA). The luciferase activity was standardized as firefly luciferase. We followed the methods of Yan et al. [17].

### 2.7. RT-qPCR

Total RNA was extracted according to the RNA extraction kit instructions. Then, reverse transcription was performed according to the instructions of the reverse transcription kit, and the transcription level of KCNQ1OT1 was detected by the RT-PCR kit with the template of the cDNA. The reaction conditions were as follows: 95°C for 10 min, 95°C for 15 s, and 60°C for 1 min; 35 cycles were amplified. We followed the methods of Yan et al. [17].

### 2.8. Western Blotting

Cells in each group were collected and lysed with cell lysate. The supernatant was collected by centrifugation at 4°C for 20 min at 10000 × *g*. The concentration of the extracted protein was determined according to the BCA kit, and protein was separated by SDS-PAGE. The protein was electrotransferred to PVDF membranes which were dyed with the Lichunhong dye solution. The membranes were cleaned three times with TBST solution for 10 min each time. The skimmed milk was sealed, and 3 mL was added to the antibody incubator. The membranes were sealed for 1 h at room temperature. Membranes were cleaned with the TSBT solution three times, for 10 min each time; incubation with the first antibody was done overnight at 4°C; incubation with the second antibody was at room temperature for 1 h. Finally, an appropriate amount of luminescent solution (A and B liquid volume was mixed) was used to incubate the membrane for 3 min; the gel imaging system was exposed, and the Quantity-One software was used to analyze the protein content. We followed the methods of Yan et al. [17].

### 2.9. Statistical Analysis

In the present research, all statistical analysis was performed using SPSS 22.0 (Chicago, IL, USA). All the data were presented as the mean + SD. Differences between the groups were compared by ANOVA (analysis of variance). Differences between the two groups were analyzed by a *t*-test. When *P* < 0.05, it indicated that the difference was statistically significant.

## 3. Results

### 3.1. KCNQ1OT1 Is Highly Expressed in Osteosarcoma Tissues and Cells, and KCNQ1OT1-siRNA Inhibits the Proliferation, Invasion, and Drug Resistance of Osteosarcoma Cells

**Figure 1:**
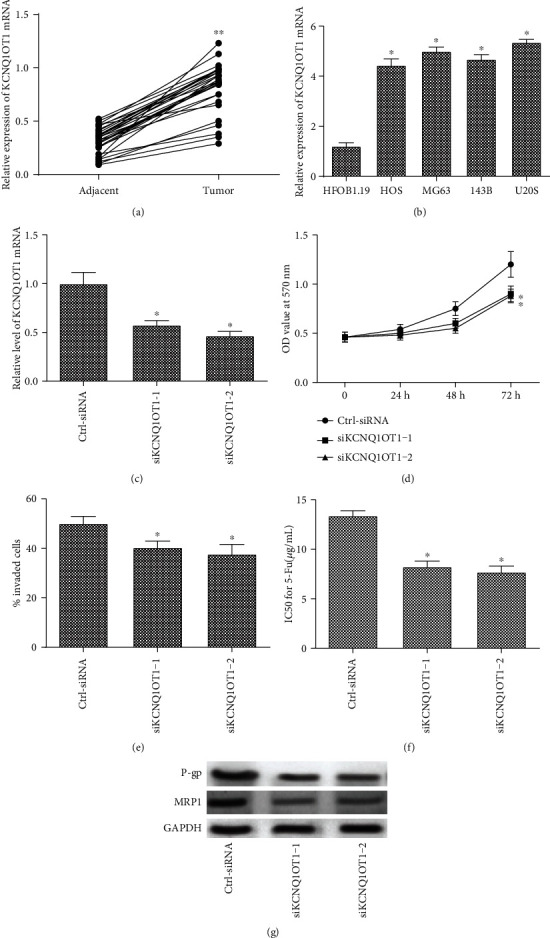
KCNQ1OT1 is highly expressed in osteosarcoma tissues and cells, and KCNQ1OT1-siRNA inhibits the proliferation, invasion, and drug resistance of osteosarcoma cells. (a) The relative level of KCNQ1OT1 mRNA in adjacent and osteosarcoma tumor tissues. (b) The expression of KCNQ1OT1 in osteoblasts (HFOB1.19) and osteosarcoma cells (HOS, MG63, 143B, and U20S). U20S cells were transfected with the control siRNA (Ctrl-siRNA group) or KCNQ1OT1-siRNA, respectively. (c) The relative mRNA expression of KCNQ1OT1 in the Ctrl-siRNA and KCNQ1OT1-siRNA groups. (d) The cell proliferation in the Ctrl-siRNA and KCNQ1OT1-siRNA groups. (e) The percentage of cell invasion in the Ctrl-siRNA and KCNQ1OT1-siRNA groups. (f) The IC50 for 5-Fu was calculated in the Ctrl-siRNA and KCNQ1OT1-siRNA group by MTT. (g) The protein expression of P-gp and MRP1 in the Ctrl-siRNA and KCNQ1OT1-siRNA groups. ^∗^Compared with the HFOB1.19 or Ctrl-siRNA group at *P* < 0.05 and ^∗∗^compared with the adjacent group at *P* < 0.01. GAPDH was used as an invariant internal control for calculating protein fold changes.

The mRNA expression of KCNQ1OT1 in tumor tissues and adjacent tissues of 30 osteosarcoma patients was analyzed. As shown in Figure 1(a), the mRNA expression of KCNQ1OT1 was increased significantly in tumor tissues than adjacent tissues (Figure 1(a)). U20S cells were transfected with the control siRNA or KCNQ1OT1-siRNA, respectively. The expression of KCNQ1OT1 was significantly decreased in the KCNQ1OT1-siRNA group than the Ctrl-siRNA group (Figure 1(c)). In addition, KCNQ1OT1-siRNA significantly inhibited cell proliferation compared with the Ctrl-siRNA group (Figure 1(d)). Meanwhile, KCNQ1OT1-siRNA downregulated the percentage of invaded cells compared with the Ctrl-siRNA group (Figure 1(e)). What is more, KCNQ1OT1-siRNA significantly decreased the IC50 for 5-Fu (Figure 1(f)). Similarly, the protein expression of P-gp and MRP1 was drastically downregulated in the KCNQ1OT1-siRNA group compared with the Ctrl-siRNA group (Figure 1(g)).

### 3.2. KCNQ1OT1 Binds to miR-129-5p Directly, and the Overexpression of LARP1 Promotes Proliferation, Invasion, and Drug Resistance of Osteosarcoma Cells

**Figure 2:**
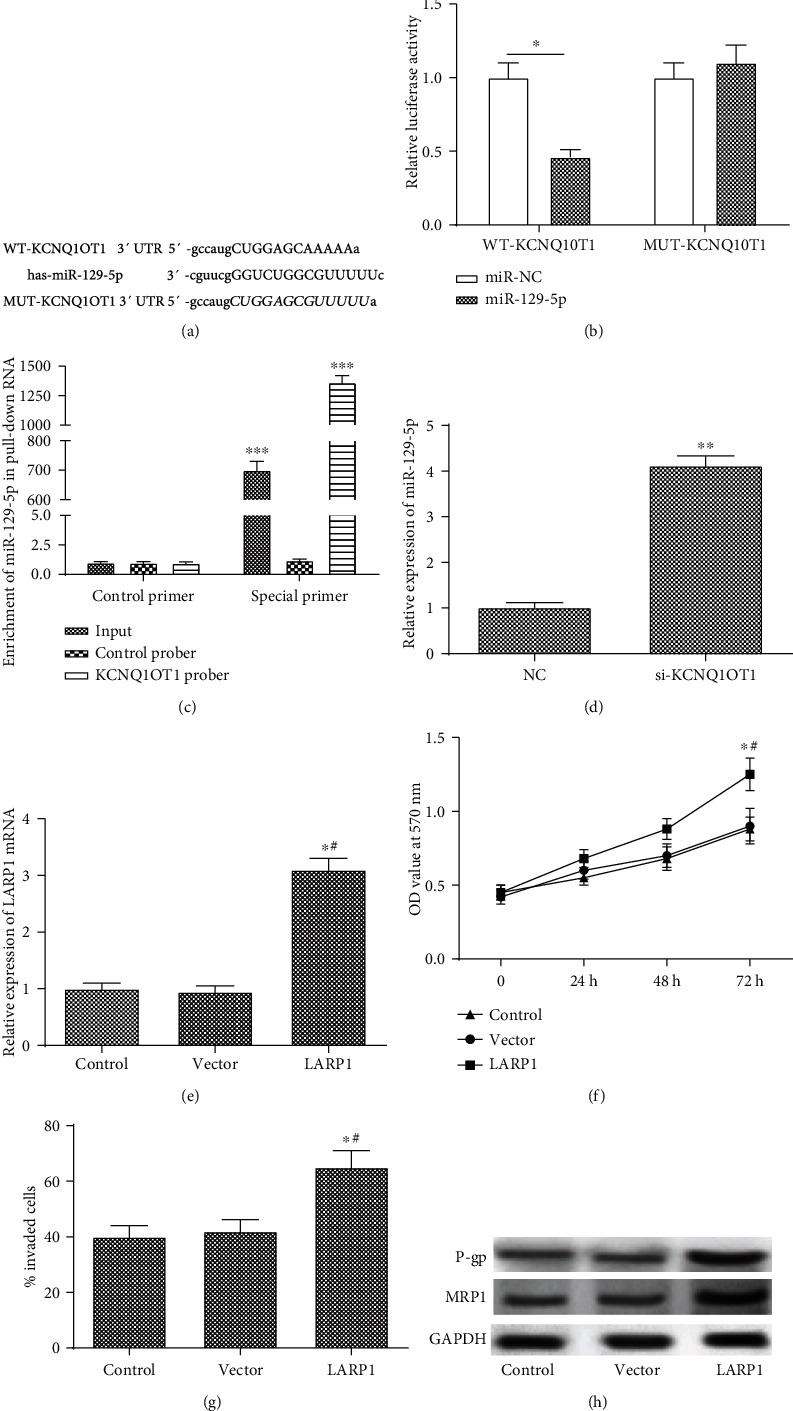
KCNQ1OT1 binds to miR-129-5p directly, and the overexpression of LARP1 promotes proliferation invasion and drug resistance of osteosarcoma cells. (a) The binding region of KCNQ1OT1 and miR-129-5p predicted by StarBase. (b) Dual luciferase reporter assay was performed to measure the activity of determination of binding activity of KCNQ1OT1 and miR-129-5p. (c) Pull-down assay was performed to measure the binding activity of KCNQ1OT1 and miR-129-5p. (d) The relative expression of miR-129-5p in the NC and siKCNQ1OT1 groups. U20S cells were transfected with the control vector (vector group) or pcDNA3.1-LARP1 (LARP1 group), respectively. (e) The relative mRNA expression of LARP1 in the vector and LARP1 groups. (f) The cell proliferation in the vector and LARP1 groups in U20S cells. (g) The percentage of cell invasion in the vector and LARP1 groups. (h) The protein expression of P-gp and MRP1 in the vector and LARP1 groups. ^∗^Compared with the control group at *P* < 0.05 and ^#^compared with the vector group at *P* < 0.05. ^∗∗^Compared with the WT-KCNQ1OT1 or NC group at *P* < 0.01 and ^∗∗∗^compared with the control primer group at *P* < 0.001. “Special primer” is the specific primer of miR-129-5p, and “control primer” is the control group. GAPDH was used as an invariant internal control for calculating protein fold changes.

To further explore the mechanism of KCNQ1OT1 in osteosarcoma cells, a luciferase report assay and pull-down experiment were performed in the research. First, we use StarBase to predict the binding motif of KCNQ1OT1 and miR-129-5p (Figure 2(a)). Luciferase analysis showed that the relative luciferase activity of transfection with miR-129-5p was significantly increased than transfection with miR-NC in the WT-KCNQ1OT1 group, while there was no significant difference in the MUT-KCNQ1OT1 group (Figure 2(b)). We also employed the pull-down assay to test if KCNQ1OT1 could pull down miR-129-5p, and a biotin-labeled-specific KCNQ1OT1 probe was used. The results showed that miR-129-5p could be coprecipitated by KCNQ1OT1 (Figure 2(c)). Meanwhile, the expression of miR-129-5p was significantly upregulated in the siKCNQ1OT group compared with the NC group (Figure 2(d)). U20S cells were transfected with the control vector (vector group) or pcDNA3.1-LARP1 (LARP1 group), respectively. The pcDNA3.1-LARP1 significantly increased the expression of LARP1 compared with the control and vector groups (Figure 2(e)). Furthermore, pcDNA3.1-LARP1 significantly promoted cell proliferation compared with the vector group in a time-dependent manner (Figure 2(f)). The percentage of invaded cells was upregulated in the pcDNA3.1-LARP1 group compared with the vector group (Figure 2(g)). What is more, the protein expression of P-gp and MRP1 was significantly increased in the LARP1 group (Figure 2(h)).

### 3.3. miR-129-5p Binds to LARP1 Directly

**Figure 3:**
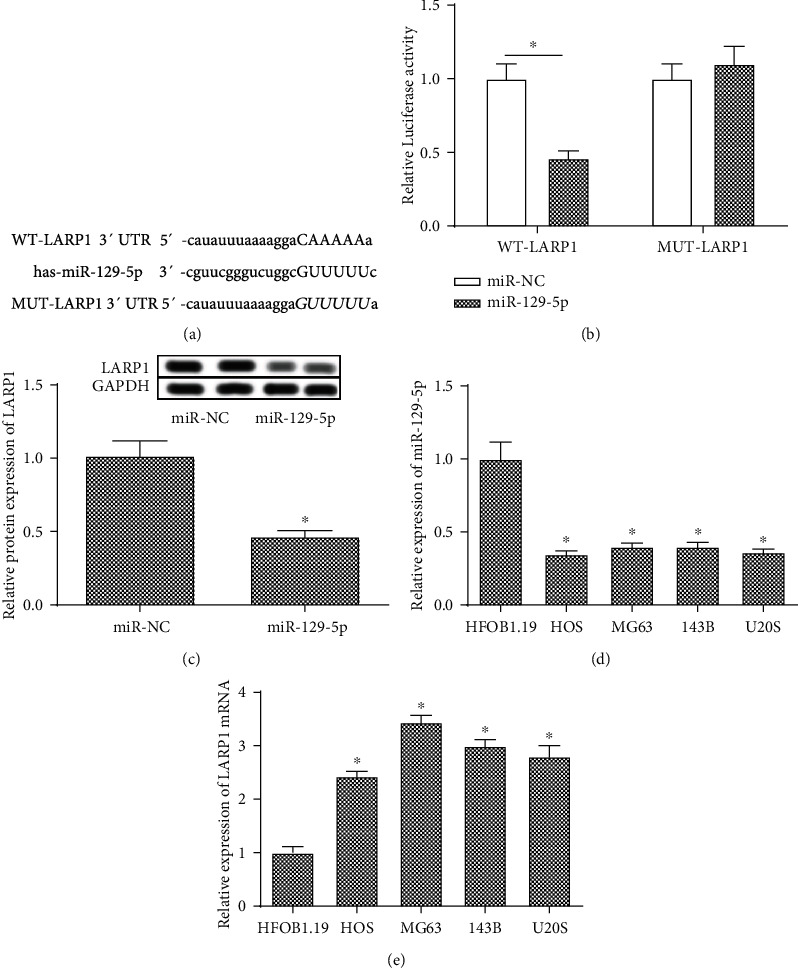
miR-129-5p binds to LARP1 directly. (a) The binding region of miR-129-5p and LARP1 predicted by StarBase. (b) Dual luciferase reporter assay was performed to measure the activity of determination of binding activity of miR-129-5p and LARP1. (c) The relative protein expression of LARP1 in the miR-NC group and the miR-129-5p group. (d) The expression of miR-129-5p in osteoblasts (HFOB1.19) and osteosarcoma cells (HOS, MG63, 143B, and U20S). (e) The mRNA expression of LARP1 in osteoblasts (HFOB1.19) and osteosarcoma cells (HOS, MG63, 143B, and U20S). ^∗^Compared with the WT-LARP1 or miR-NC group at *P* < 0.05. GAPDH was used as an invariant internal control for calculating protein fold changes.

As shown in Figure 3(a), we use StarBase to predict the binding motif of miR-129-5p and LARP1 (Figure 3(a)). The results showed that the relative luciferase activity of transfection with miR-129-5p was significantly increased compared with transfection with miR-NC in the WT-LARP1 group (Figure 3(b)). Furthermore, the protein expression of LARP1 was significantly decreased after transfection with miR-129-5p mimic (Figure 3(c)). What is more, the expression of miR-129-5p in osteosarcoma cell lines was significantly downregulated compared with the normal osteoblast cell lines (Figure 3(d)). And the mRNA expression of LARP1 in osteosarcoma cell lines was significantly increased than normal osteoblast cell lines (Figure 3(e)).

### 3.4. KCNQ1OT1 Promotes Proliferation, Invasion, and Drug Resistance by Regulating miR-129-5p-Mediated LARP1 in Osteosarcoma Cells

**Figure 4:**
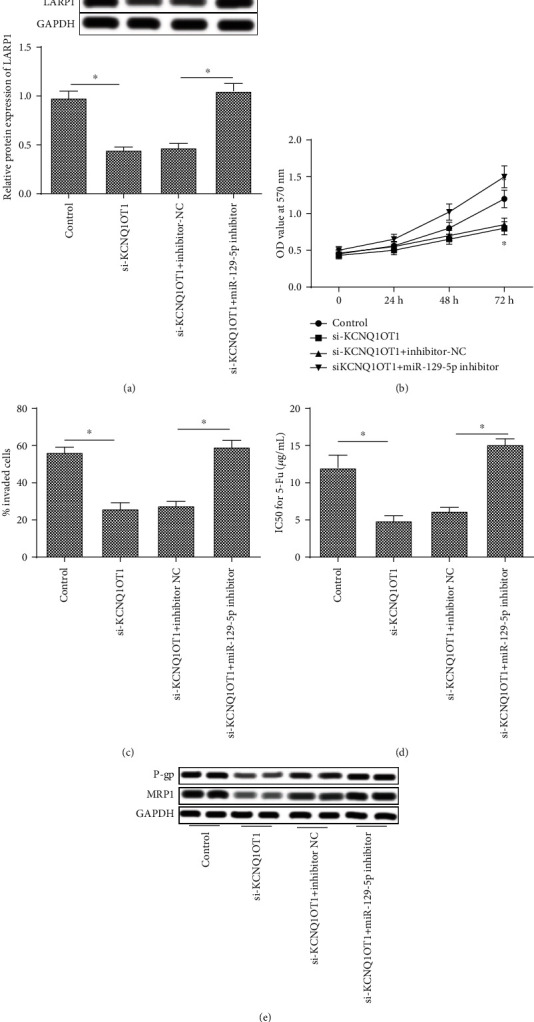
KCNQ1OT1 promotes proliferation, invasion, and drug resistance by regulating miR-129-5p-mediated LARP1 in osteosarcoma cells. U20S cells were transfected with the control vector (control group), KCNQ1OT1-siRNA (siBCLAF1 group), KCNQ1OT1-siRNA+control inhibitor (siBCLAF1+inhibitor-NC group), or KCNQ1OT1-siRNA+miR-129-5p inhibitor (siKCNQ1OT1+miR-129-5p inhibitor group), respectively. (a) The protein expression of LARP1 in the control group, siBCLAF1 group, siBCLAF1+inhibitor-NC group, and siKCNQ1OT1+miR-129-5p-inhibitor group. (b) The cell proliferation in the control group, siBCLAF1 group, siBCLAF1+inhibitor-NC group and siKCNQ1OT1+miR-129-5p inhibitor group. (c) The percentage of cell invasion in the control group, siBCLAF1 group, siBCLAF1+inhibitor-NC group, and siKCNQ1OT1+miR-129-5p inhibitor group. (d) The protein expression of P-gp and MRP1 in the control group, siBCLAF1 group, siBCLAF1+inhibitor-NC group, and siKCNQ1OT1+miR-129-5p inhibitor group. (e) The IC50 for 5-Fu was calculated in the control group, siBCLAF1 group, siBCLAF1+inhibitor-NC group, and siKCNQ1OT1+miR-129-5p inhibitor group. ^∗^Compared with the control group or siBCLAF1+inhibitor-NC group at *P* < 0.05. GAPDH was used as an invariant internal control for calculating protein fold changes.

U20S cells were transfected with the control vector (control group), KCNQ1OT1-siRNA (siKCNQ1OT1 group), siKCNQ1OT1+NC inhibitor (siKCNQ1OT1+inhibitor-NC group), or siKCNQ1OT1+miR-129-5p inhibitor (siKCNQ1OT1+miR-129-5p inhibitor group), respectively. As shown in Figure 4(a), the protein expression of LARP1 was downregulated significantly in the siKCNQ1OT1 group, which was reversed by the miR-129-5p inhibitor (Figure 4(a)). The cell proliferation was increased in the siKCNQ1OT1+miR-129-5p inhibitor group compared with the siKCNQ1OT1+inhibitor-NC group (Figure 4(b)). In addition, cotransfection with siKCNQ1OT1 and miR-129-5p inhibitor upregulated the percentage of invaded cells compared with the siKCNQ1OT1+inhibitor-NC group (Figure 4(c)). The IC50 for 5-Fu was decreased significantly in the siBCLAF1 group and increased in the siKCNQ1OT1+miR-129-5p inhibitor group (Figure 4(d)). Similarly, the protein expression of P-gp and MRP1 was decreased in the siKCNQ1OT1 group compared with the control group, and the miR-129-5p inhibitor reversed the inhibitory effect of siKCNQ1OT1 on the protein expression of P-gp and MRP1 (Figure 4(e)).

## 4. Discussion

In the present study, we focused on the effect of KCNQ1OT1 on cell proliferation, invasion, and drug resistance in osteosarcoma as well as the special regulatory mechanisms. The expression of KCNQ1OT1 was upregulated in tumor tissues. The knockdown of KCNQ1OT1 inhibits the cell proliferation, invasion, and drug resistance by targeting miR-129-5p. Further studies have found that LARP1 is a direct target of miR-129-5p and promotes the proliferation, invasion, and drug resistance of osteosarcoma cells. Therefore, the knockdown of KCNQ1OT1 inhibits the occurrence and development of osteosarcoma via regulating the miR-129-5p/LARP1 axis.

KCNQ1OT1 is an lncRNA highly expressed in a variety of malignant tumors, such as colorectal, lung, tongue, and liver cancers, adrenal cortical tumors, and glioma [18–20]. It reported that the expression of KCNQ1OT1 in osteosarcoma tissues was significantly increased than adjacent tissues, and the high expression of KCNQ1OT1 may promote the development of osteosarcoma [21]. Similarly, our study found that the expression of KCNQ1OT1 in osteosarcoma was also upregulated which is consistent with previous studies. Functional studies have found that KCNQ1OT1 could promote cell proliferation, migration, and invasion and inhibit cell apoptosis [6, 22]. In addition, KCNQ1OT1 was also found to be associated with drug resistance. Ma et al. reported that the level of KCNQ1OT1 in chemically insensitive TSCC tissues was significantly increased than chemically sensitive TSCC tissues. In addition, KCNQ1OT1 promoted the proliferation of TSCC in vitro and in vivo and endowed TSCC with cisplatin-induced apoptosis resistance [19]. Our results suggest that KCNQ1OT1-siRNA not only inhibits cell proliferation and invasion but also, more importantly, inhibits drug resistance for 5-Fu. It is suggested that KCNQ1OT1 is an oncogene that promotes malignant progression of cancer cells [18]. Therefore, KCNQ1OT1 may be a new biomarker for cancer detection.

Prediction software analysis showed that miR-129-5p was the downstream target gene of KCNQ1OT1. It is reported that miR-129-5p has been identified as a human tumor suppressor [23]. The expression of miR-129-5p in osteosarcoma cells is lower than that in normal tissues and cells, and the increase of miR-129-5p may be mediated by demethylation and inhibit the migration and invasion of OS cells by targeting VCP in OS [24]. miR-129-5p, a downstream target gene of lncRNA MALAT1, targeting regulates downstream transcription factor HMGB1 and participates in OS cell proliferation and tumor progression [25]. As we expected, our results showed that miR-129-5p inhibited the malignant progression of osteosarcoma cells, including the cell proliferation, migration, and invasion, while inhibiting cell drug resistance.

LARP1 is regarded as an oncogene. Studies have reported that the expression of LARP1 in ovarian cancer cells is significantly upregulated, which is necessary for cancer cell survival and chemotherapy resistance [13]. The expression of LARP1 in NSCLC cells was upregulated compared with the normal control cells, and the downregulation of LARP1 inhibited migration, invasion, and proliferation of NSCLC cells and the tumorigenicity of H520 cells [26]. Likewise, the expression of LARP1 was upregulated in cervical cancer and NSCLC, and its expression was associated with disease progression and adverse prognosis. Likewise, in our study, LARP1, as a downstream target gene of miR-129-5p, promotes the proliferation, invasion, and drug resistance of osteosarcoma cells, which is consistent with previous studies.

In summary, our current study reveals that the knockdown of KCNQ1OT1 inhibits the occurrence and development of osteosarcoma by regulating the miR-129-5p/LARP1 axis, which suggests that KCNQ1OT1 might be a new biomarker related to proliferation and drug resistance of osteosarcoma. In the future, the search for a deep and reasonable mechanism for the role of KCNQ1OT1 will help us to understand its function more comprehensively and finally find a new method for the treatment of human malignant tumor.

## Data Availability

Data is available on request.

## Supplementary Materials

Supplementary Materials Graphical picture.

## References

[1] Lindsey B. A., Markel J. E., Kleinerman E. S. (2017). Osteosarcoma overview. *Rheumatology and Therapy*.

[2] Xie X., Li Y.-S., Xiao W.-F. (2017). MicroRNA-379 inhibits the proliferation, migration and invasion of human osteosarcoma cells by targetting EIF4G2. *Bioscience Reports*.

[3] Liu D., Zhang C., Li X., Zhang H., Pang Q., Wan A. (2018). MicroRNA-567 inhibits cell proliferation, migration and invasion by targeting FGF5 in osteosarcoma. *EXCLI Journal*.

[4] Jiang H., Wang X., Miao W., Wang B., Qiu Y. (2017). CXCL8 promotes the invasion of human osteosarcoma cells by regulation of PI3K/Akt signaling pathway. *APMIS*.

[5] Liz J., Esteller M. (2016). lncRNAs and microRNAs with a role in cancer development. *Biochimica et Biophysica Acta*.

[6] Sunamura N., Ohira T., Kataoka M. (2016). Regulation of functional KCNQ1OT1 lncRNA by *β*-catenin. *Scientific Reports*.

[7] Seiji N., Kazuhiro M., Makiko M. (2010). Expression profile of *LIT1/KCNQ1OT1* and epigenetic status at the KvDMR1 in colorectal cancers. *Cancer Science*.

[8] Wan J., Huang M., Zhao H. (2013). A novel tetranucleotide repeat polymorphism within KCNQ1OT1 confers risk for hepatocellular carcinoma. *Dna & Cell Biology*.

[9] Zhang P., Li J., Song Y., Wang X. (2017). miR-129-5p inhibits proliferation and invasion of chondrosarcoma cells by regulating SOX4/Wnt/*β*-catenin signaling pathway. *Cellular Physiology and Biochemistry*.

[10] Jiang Z., Wang H., Li Y. (2016). miR-129-5p is down-regulated and involved in migration and invasion of gastric cancer cells by targeting interleukin-8. *Neoplasma*.

[11] Yu X., Luo L., Wu Y. (2013). Gastric juice miR-129 as a potential biomarker for screening gastric cancer. *Medical Oncology*.

[12] Wu J., Qian J., Li C. (2014). miR-129 regulates cell proliferation by downregulating Cdk6 expression. *Cell Cycle*.

[13] Hopkins T. G., Mura M., Al-Ashtal H. A. (2016). The RNA-binding protein LARP1 is a post-transcriptional regulator of survival and tumorigenesis in ovarian cancer. *Nucleic Acids Research*.

[14] Mura M., Hopkins T. G., Michael T. (2015). LARP1 post-transcriptionally regulates mTOR and contributes to cancer progression. *Oncogene*.

[15] Lahr R. M., Mack S. M., Héroux A. (2015). The La-related protein 1-specific domain repurposes HEAT-like repeats to directly bind a 5'TOP sequence. *Nucleic Acids Research*.

[16] Merret R., Descombin J., Juan Y. T. (2013). XRN4 and LARP1 are required for a heat-triggered mRNA decay pathway involved in plant acclimation and survival during thermal stress. *Cell Reports*.

[17] Yan K., Xu X., Wu T. (2019). Knockdown of PYCR1 inhibits proliferation, drug resistance and EMT in colorectal cancer cells by regulating STAT3-mediated p38 MAPK and NF-*κ*B signalling pathway. *Biochemical and Biophysical Research Communications*.

[18] Gong W., Zheng J., Liu X. (2017). Knockdown of long non-coding RNA KCNQ1OT1 restrained glioma cells’ malignancy by activating miR-370/CCNE2 axis. *Frontiers in Cellular Neuroscience*.

[19] Zhang S., Ma H., Zhang D. (2018). LncRNA KCNQ1OT1 regulates proliferation and cisplatin resistance in tongue cancer via miR-211-5p mediated Ezrin/Fak/Src signaling. *Cell Death & Disease*.

[20] Ren K., Xu R., Huang J., Zhao J., Shi W. (2017). Knockdown of long non-coding RNA KCNQ1OT1 depressed chemoresistance to paclitaxel in lung adenocarcinoma. *Cancer Chemotherapy and Pharmacology*.

[21] Zhang C., Du S., Cao L. (2018). Long non-coding RNA KCNQ1OT1 promotes osteosarcoma progression by increasing *β*-catenin activity. *RSC Advances*.

[22] Tano K., Akimitsu N. (2012). Long non-coding RNAs in cancer progression. *Frontiers in Genetics*.

[23] Zhang H., Cai Y., Zheng L., Zhang Z., Lin X., Jiang N. (2018). Long noncoding RNA NEAT1 regulate papillary thyroid cancer progression by modulating miR-129-5p/KLK7 expression. *Journal of Cellular Physiology*.

[24] Long X. H., Zhou Y. F., Peng A. F. (2015). Demethylation-mediated miR-129-5p up-regulation inhibits malignant phenotype of osteogenic osteosarcoma by targeting Homo sapiens valosin-containing protein (VCP). *Tumour Biology*.

[25] Liu K., Huang J., Ni J. (2017). MALAT1 promotes osteosarcoma development by regulation of HMGB1 via miR-142-3p and miR-129-5p. *Cell Cycle*.

[26] Xu Z., Xu J., Lu H. (2017). LARP1 is regulated by the XIST/miR-374a axis and functions as an oncogene in non-small cell lung carcinoma. *Oncology Reports*.

