# Supplementary figures and images for: Knockdown of KCNQ1OT1 Inhibits Proliferation, Invasion, and Drug Resistance by Regulating miR-129-5p-Mediated LARP1 in Osteosarcoma
Source: Biomed Res Int. 2020 Sep 4;2020:7698767. doi: 10.1155/2020/7698767 (PMC7487100; doi:10.1155/2020/7698767)

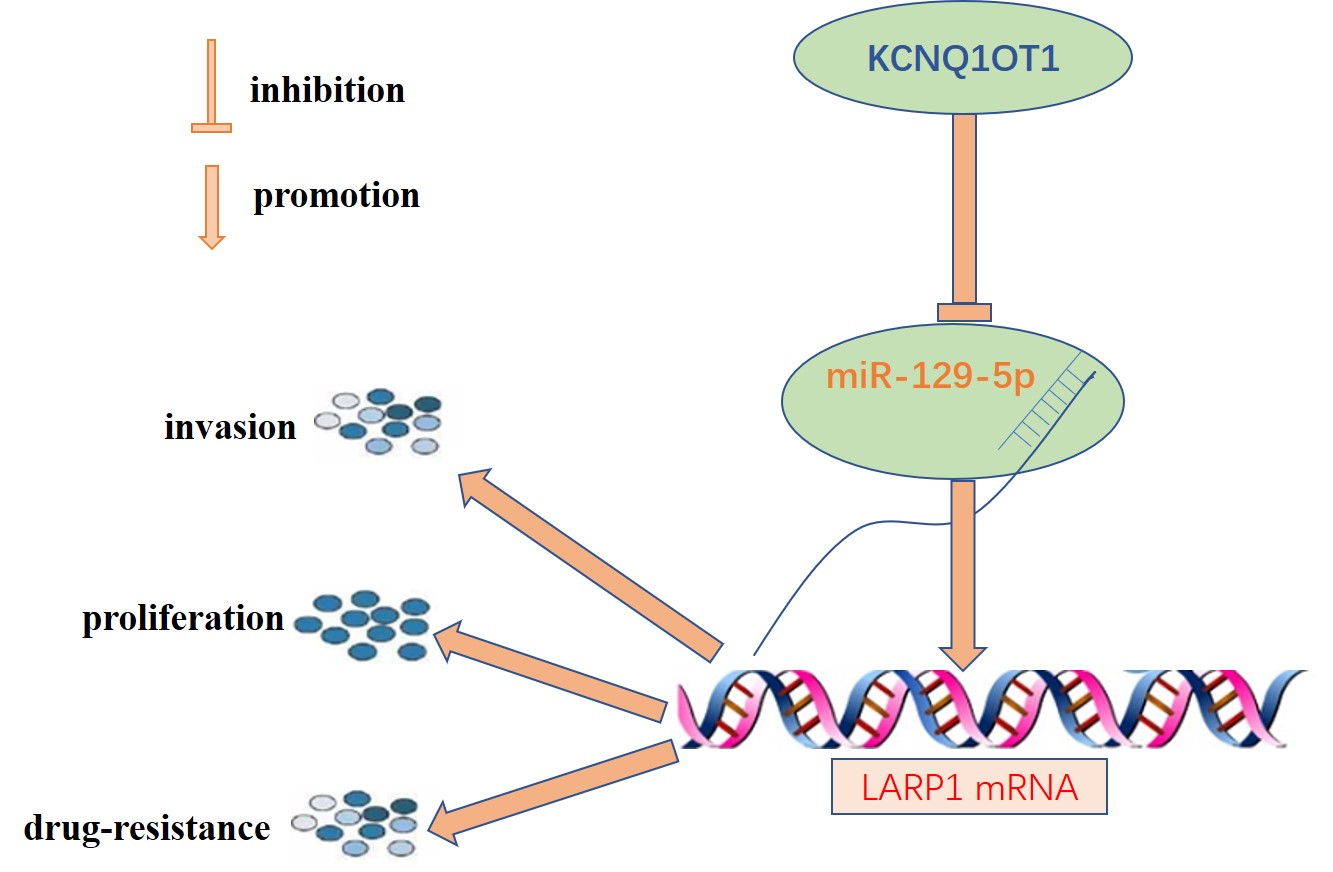

Supplement: Supplementary Materials — Graphical picture. [file 7698767.f1.jpg]
